# ASSOCIATION AND INTERACTION BETWEEN RESILIENCE AND PSYCHOLOGICAL DISTRESS IN DISABLED ELDERLY–CAREGIVER DYADS

**DOI:** 10.1093/geroni/igae098.2687

**Published:** 2024-12-31

**Authors:** Anni Wang

**Affiliations:** Fudan University, Shanghai, Shanghai, China (People’s Republic)

## Abstract

**Objectives:**

The aim of this study was to explore the interaction between resilience and psychological distress in disabled elderly-caregiver.

**Methods:**

A total of 246 homebound disabled elderly and their family caregivers from five provinces of China were investigated by convenience sampling using the Chinese version of the Resilience Scale and the Distress Thermometer and Problem List. Data analysis using the actor - partner interdependence model (APIM) modelling.

**Results:**

The effect of resilience on psychological distress is a mixed pattern. With regard to the actor effect, the resilience of the disabled elderly and caregivers is negatively related to their own psychological distress, respectively. Regarding the partner effect, the resilience of the disabled elderly was negatively associated with caregiver psychological distress, and the resilience of the caregiver was negatively associated with psychological distress of the disabled elderly.

**Conclusion:**

There is dyadic interaction between disabled elderly and their caregivers, suggesting that resilience can relieve not only one’s own psychological stress but also that of others to some extent. Families of disabled elderly need to focus on both the older person and the caregiver, taking advantage of resilience in a focused way to reduce psychological distress and improve the dyadic physical and mental health. Health professionals can design dyadic interventions which are focuses on resilience and its dyadic interaction, and effectively leverage the resilience advantages of the dyad based on the individual’s level of psychological distress, to improve the mental health of the elderly with disabilities and their caregivers.

